# Microstructure, Mechanical Properties, and in Vitro Corrosion Behavior of Biodegradable Zn-1Fe-xMg Alloy

**DOI:** 10.3390/ma13214835

**Published:** 2020-10-29

**Authors:** Penghao Xue, Minglong Ma, Yongjun Li, Xinggang Li, Jiawei Yuan, Guoliang Shi, Kaikun Wang, Kui Zhang

**Affiliations:** 1 State Key Laboratory of Nonferrous Metals and Processes, GRIMAT Engineering Institute Co., Ltd., Beijing 100088, China; b20170187@xs.ustb.edu.cn (P.X.); maminglong@grinm.com (M.M.); lyj@grinm.com (Y.L.); lxg1218@grinm.com (X.L.); yuanjiawei@grinm.com (J.Y.); shigl@grinm.com (G.S.); 2 School of Materials Science and Engineering, University of Science & Technology Beijing, Beijing 100083, China; kkwang@mater.ustb.edu.cn; 3 GRIMAT Engineering Institute Co., Ltd., Beijing 101407, China; 4 General Research Institute for Nonferrous Metals, Beijing 100088, China

**Keywords:** Zn-Fe-Mg alloy, mechanical properties, electrochemical, corrosion model

## Abstract

Zinc (Zn), one of the promising candidates for biodegradable implant materials, has excellent biocompatibility and biodegradability. In this study, as-cast Zn1FexMg (x ≤ 1.5 wt %) alloys were prepared to systematically explore the effects of magnesium (Mg) alloying on their microstructures, mechanical properties, and biodegradability. The microstructure of Zn1FexMg alloy consisted of Zn matrix, Zn + Mg_2_Zn_11_ eutectic structure, and FeZn_13_ phase. The addition of Mg not only promoted grain refinement of the alloy, but also improved its mechanical properties. The results of immersion tests showed that the addition of Mg accelerated microcell corrosion between different phases, and the modeling of the corrosion mechanism of alloys in simulated body fluid (SBF) solution was discussed to describe the interaction between different phases in the corrosion process. Zn1Fe1Mg possessed superior comprehensive mechanical properties and appropriate corrosion rate, and the values for hardness, tensile strength, yield strength, elongation, and corrosion rate were 105 HB, 157 MPa, 146 MPa, 2.3%, and 0.027 mm/a, respectively, thus revealing that Zn1Fe1Mg is a preferred candidate for biodegradable implant material.

## 1. Introduction

In addition to having excellent biocompatibility, good biodegradable metal materials should provide adequate support during the cytothesis and the remodeling of tissue, for example during bone and vascular remodeling. Once bone and vascular remodeling have been accomplished, the degradation should occur as quickly as possible to avoid stress shielding or secondary blockage [1,2]. Early studies mainly focused on pure Iron (Fe) and Magnesium (Mg) and their alloys due to their excellent biocompatibility. However, Fe-based alloys possess a low degradation rate, and their harmful corrosion products impair the integrity of the arterial wall [3,4,5,6,7]. Mg-based alloys have unsatisfactory mechanical properties and the accelerated corrosion rates in physiological environments predispose them towards premature loss of mechanical integrity as well as gas embolism due to the harmful corrosion-linked formation of hydrogen gas [8,9,10,11]. Thus, researchers are eager to identify other types of alloys, which can replace the above-mentioned alloys, among them Zinc (Zn) and Zn-based alloys.

Zn element plays an important role in the biological functions of the human body because it is involved in all aspects of cell metabolism. It also supports immune function, protein and DNA synthesis, and wound healing [12,13,14,15]. Zn exhibits high chemical activity, with an electrode potential value between that of Mg and Fe. Thus, Zn has received attention in recent years due to its good performance and biocompatibility. In animal experiments, pure Zn implants placed in porcine arteries have shown good biocompatibility [13]. However, a previous study showed that pure Zn has very low mechanical strength. Under casting and forging conditions, the ultimate tensile strength (UTS) of pure Zn is only 20 MPa and 120 MPa [16,17]. Current efforts to counteract the inherent disadvantageous properties of Zn alloys are largely focused on alloying and improved processing conditions. The Zn-Fe base alloys in particular have attracted great attention as iron is one of the most abundant nutritional elements in the human body [18,19,20]. As observed in the Zn-Fe binary phase diagram [21], a peritectic reaction occurs during solidification, which forms primary Fe-Zn binary phases. The needle-shaped Fe-Zn phase and the Zn matrix form a structure similar to “reinforced concrete”, which significantly improves the mechanical properties. The Fe-Zn phase and the Zn matrix will also cause microcell corrosion, which accelerates the degradation rate of implants made from Zn alloys [18]. Alon Kafri et al. implanted a cylindrical disk made of as-cast Zn-2wt % Fe alloy into the back midline of male Wistar rats for 24 weeks. Results of well-being, hematological testing, and histological analysis in rats indicated that the in-vivo behavior of Zn-2% Fe implants was satisfactory [19]. However, the elongation of the as-cast Zn-1.3Fe alloy is even less than 2% [17]. The coarse Fe-Zn phases weaken the plasticity and make alloys with high brittleness, which limits the potential application prospect.

In order to reduce the brittleness of the alloy, the amount of Fe added must be restricted within 1 wt %. Thus, in order to further optimize the comprehensive properties of Zn-Fe alloy, Mg element was considered to be a microalloying element based on the following reasons. Firstly, Mg is a commonly added element in Zn-based biodegradable alloy, and Zn-Mg binary alloys have already been extensively explored [22,23,24,25]. Secondly, the addition of a small amount of Mg to pure Zn helps to form eutectic structures (Mg_2_Zn_11_ + Zn) at the grain boundaries, hindering the grain growth, thus enhancing the mechanical properties. Thirdly, the Mg_2_Zn_11_ phase may act as an anodic phase to Zn matrix and FeZn_13_ phase, causing accelerated corrosion via microcell corrosion, because the high potential difference among Mg_2_Zn_11_, FeZn_13_, and Zn may cause microcell corrosion. Some scholars improve the properties of the alloy by adding elements such as Ca, Sr, Al to Zn-Mg [26,27,28]. Some of these elements seem to play the same role as Fe. For example, CaZn_13_ strengthens the mechanical properties of Zn-Mg-Ca and promote its degradation. However, Ca and Sr are very expensive, and they are so active that the preparations are more complicated. Al is not the first element in the alloying of degradable alloys because of its negative effects on the human body. Lastly, due to the “reinforced concrete” structure of alloy, the existence of the needle-like Fe-Zn phase ensures that preferential corrosion cracking at the grain boundaries does not cause premature failure of the implants. It may ensure the ability of long-term support to blood vessels or bones during their lifespan. In this study, in order to comprehend mechanical properties and accelerate the degradation rate of Zn-Fe alloy as a biodegradable candidate material, Zn-1Fe-xMg (x = 0.5−1.5 wt %) alloys were designed, with an aim to explore the influence of Mg on the microstructure, mechanical properties, and corrosion characteristics of the biodegradable Zn-1Fe-Mg. A corrosion model of the alloy was established to determine the interaction between different phases of in vitro corrosion behavior of alloys to provide basic guidance for Mg alloying in biodegradable Zn-Fe alloys.

## 2. Experimental

### 2.1. Material Preparation

Zn1FexMg alloys with different Mg contents (0.5, 1.0, and 1.5 wt %) were prepared by gravity casting under a protective atmosphere of Argon (Ar) and tetrafluoroethane R-134a to inhibit the oxidation of Mg. Boron carbide was sprayed on the surface of the cylindrical cast iron crucibles with a size of φ100 × 200 mm to prevent the surface reaction with Zn during the smelting process. Zn ingots (purity > 99.99%) were melted in the crucible at 500 °C. Heating was continued until 650 °C was reached, and the Mg ingots (purity > 99.90%) were pressed into the bottom of the solution with a bell jar. After the Mg ingots melted, the iron powder (purity > 99.90%) was pressed into the melt with a bell jar. The melt was homogenized by intense mechanical stirring, and then it was poured into a permanent mold. The obtained Zn-1Fe-xMg alloys were cut into samples of different sizes for follow-up testing. The chemical compositions of samples tested by Inductively Coupled Plasma Optical Emission Spectrometry (ICP-OES, Agilent 725, Agilent, Santa Clara, CA, USA) are shown in Table 1. As the reference burning loss rates of the alloying elements were not consistent with the actual burning loss rate of the alloying elements, there was a difference between the calculated and actual components of the alloy.

### 2.2. Material Characterization

All samples used for microscopic observation were mechanically polished to 1 μm and etched with dilute nitric acid solution. The microstructures of Zn1FexMg alloys were observed using an optical microscope (Axiovert-200MAT, Zeiss, Jena, Germany) and scanning electron microscopy (SEM, JSM-7610F, Jeol, Tokyo, Japan) equipped with energy dispersive spectroscopy (EDS, AMETEK EDAX, Berwyn, PA, USA). Moreover, the X-ray diffractometer (XRD, Xpert Pro, PANalytical, Amsterdam, Netherlands) with Cu-Kα radiation was used to identify the phases in alloys. The scan angle range was 10–90°, the diffraction parameter was 40 kV/30 mA, and the scan rate was 2°/min.

The mechanical properties of the samples were tested according to the ASTM-E8/E8m-11 standard [10] with a crosshead speed of 1 mm/min on the universal material test machine (SANS, CMT5504, USA). The Brinell hardness value of polished samples was measured using an Brinell hardness tester (XHB-3000, Wuhan, China). The applied load was 3000 Kgf and the residence time was 15 s.

### 2.3. In Vitro Corrosion Assay

The electrochemical impedance spectroscopy (EIS) test and the potentiodynamic polarization test were conducted in the SBF solution (PH1820-phygene) at 37 °C using an electrochemical workstation (CS2350H). The ionic compositions (mM) of SBF were 142.0 Na^+^, 5.0 K^+^, 1.5 Mg^2+^, 2.5 Ca^2+^, 103.0 Cl^−^, 10.0 HCO^−^_3_, 1.0 HPO^2−^_4_, 0.5 SO^2−^_4_. The Zn1FexMg alloy was the working electrode, the platinum sheet was the counter electrode, and Ag/AgCl/ in saturated KCl electrode was the reference electrode. The sample was wrapped in epoxy resin, and only a surface area of 1 cm^2^ was exposed to the SBF solution during the electrochemical test. In order to obtain a relatively stable potential value, the open-circuit potential (OCP) test was performed before the EIS and the polarization experiments for 1800 s. The EIS measurements were carried out between 0.01 Hz and 100 kHz at 10 mV amplitude. The potentiodynamic polarization tests were conducted using a scan rate of 1 mV/s and a scan range from −1600 to −400 mV. The corrosion current density was calculated by extrapolating the polarization curve, and the corrosion rate was calculated according to the ASTM-G102-89 standard [29]. The surface morphology of the specimens after electrochemical tests was further observed by SEM.

The immersion tests were performed in SBF solution according to the ASTM G31-12a standard [30]. The rectangular parallelepiped samples with an exposed area of 3 cm^2^ were immersed in a 60 mL SBF solution at 37 °C for 720 h and the SBF solution was renewed every 48 h. Then, the surface morphology was observed using SEM and corrosion products were identified by EDS. The corrosion products of tested samples were removed by means of immersion in an aqueous solution containing CrO_3_. Then the corrosion rate of the alloy was calculated.

## 3. Results and Discussion

### 3.1. Surface Morphologies and Phase Structure

**Figure 1:**
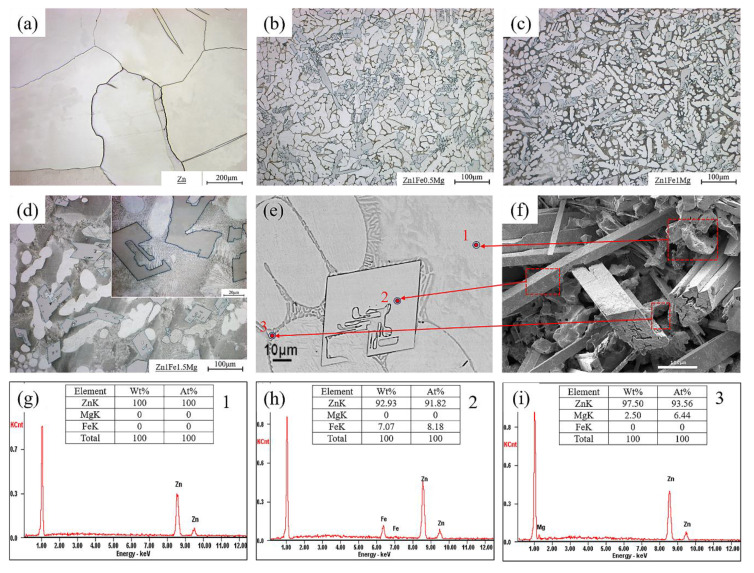
Micrographs of alloys with different Mg contents and results of energy dispersive spectroscopy (EDS) analysis: (**a**)cast Zn, (**b**) Zn-1.0Fe-0.5Mg, (**c**) Zn-1.0Fe-1.0Mg, and (**d**) Zn-1.0Fe-1.5Mg, (**e**) morphology of the Zn1Fe0.5Mg alloy in the scanning electron microscopy (SEM) image; (**f**) the surface morphology of Zn1Fe0.5Mg after removing the corrosion products; (**g**–**i**) the EDS composition analysis of the points corresponding to the positions 1, 2, and 3 of (**e**), respectively.

**Figure 2:**
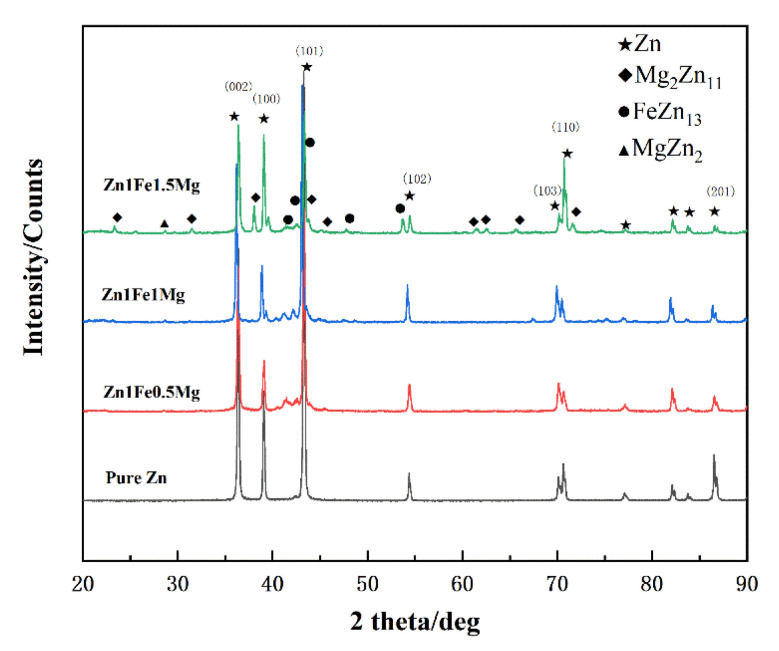
The comparison of XRD patterns of Zn1FexMg alloys.

Optical micrographs of the microstructures of cast samples are shown in Figure 1. The grains in pure Zn were large and the grain size exceeded 300 μm, as shown in Figure 1a. Figure 1b–d show the microstructures of Zn1FexMg alloys, which consisted of primary Zn dendrites, FeZn_13_ acicular structure, and eutectics along the grain boundaries. EDS analyses (see Figure 1g–i) of alloys showed that the eutectics (dark lamellar areas) consisted of Zn and Zn-Mg intermetallic compound, and the needle-like structures (white areas) were Fe-Zn intermetallic compounds, as confirmed by XRD in Figure 2. The FeZn_13_ phase nucleated and grew before the other phases, according to the Zn-Mg and Zn-Fe phase diagrams [25]. Thus, FeZn_13_ may be the nucleated particle of other phases. Optical micrographs did not reflect the true morphology and distribution of the FeZn_13_ phase. These parameters could be determined by the surface morphology of the corrosion pits in the Zn1Fe0.5Mg alloy after immersion in SBF solution for 45 days, as shown in Figure 1f. The eutectic structures at the location of the corrosion pits were corroded completely because they have a more negative potential as the anode, leaving the deeper pits shown in Figure 1f. Most of the Zn matrix is corroded, and the remaining part is shown in Figure 1f as small bumps. The FeZn_13_ phase had a more positive potential as the cathode to be protected, thus exposing the uncorroded FeZn_13_ phase, which showed a prismatic shape, with a length ranging between 100 μm and 500 μm, and it interlaced with the matrix. Corrosion properties of alloys are discussed in Section 3 of this chapter. As the Mg content increased, the volume fraction of the eutectic structure of the alloy increased, as shown in Figure 1b–d. The volume fraction of the eutectic structure of Zn1Fe1.5Mg was higher than that of the other samples, and it was clearly seen that the eutectic structure contained a submicron-sized mixture of Zn and Mg_2_Zn_11_ (see Figure 1d).

The Zn grain size of Zn1FexMg alloys was substantially smaller than that of pure Zn. The average size of Zn grains increased initially and then decreased with an increase in Mg content; the Zn grain size of Zn1Fe1Mg alloys reached a minimum value of 22.08 μm, as shown in Table 2. The reason behind Zn grain growth in Zn1Fe1.5Mg was that the high-volume fraction of the eutectic structure caused segregation of Zn during solidification (see Table 2). The section size of FeZn_13_ increased significantly, from 28.23 to 38 μm. According to the Zn-Fe phase diagram [25], there was a wide temperature range for the formation of FeZn_13_ by the peritectic reaction and it extended from 530 °C to room temperature. The size of FeZn_13_ was affected by the solidification cooling rate and hindrance of the Zn matrix. When the cooling rate was constant, the volume fraction of the eutectic structure with a low melting point increased and it reduced the hindering effect of the matrix on FeZn_13_, which can grow over a wider temperature range. The above reason can explain the growth of FeZn_13_ particles.

XRD tests were performed to investigate the effect of different Mg contents on the phases of Zn1FexMg alloys, as shown in Figure 2. The tested samples had obvious characteristic peaks of Zn, Mg_2_Zn_11_, FeZn_13_, and MgZn_2_. With an increase in Mg, the peaks of Mg_2_Zn_11_ increased significantly. When the Mg content was increased to 1.5 wt %, significantly more peaks of the eutectic structure were detected.

### 3.2. Mechanical Properties

#### 3.2.1. Hardness Test

**Figure 3:**
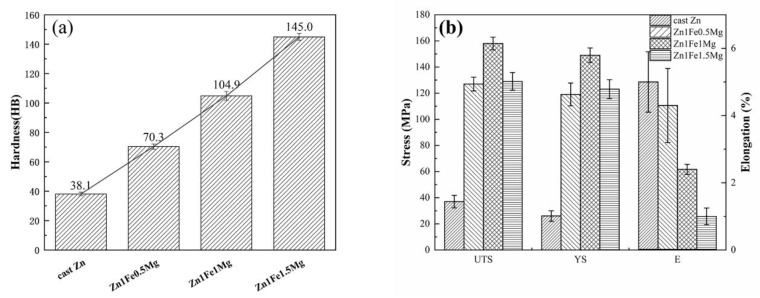
Microhardness (**a**) and tensile properties (**b**) of alloys with different Mg contents.

The hardness values of the alloys with different Mg contents are shown in Figure 3a. It was observed that hardness of Zn1FexMg alloys increased with an increase in Mg content, from approximately 38 HB for pure Zn up to 145 HB for the Zn1Fe1.5Mg alloy, and there was an increase of 280.5%. This behavior can be attributed to the significantly increasing fraction of the hard Mg_2_Zn_11_ + Zn eutectic microstructure with an increase in Mg content (see Figure 1). The alloys exhibited the characteristics, such as soft matrix and hard spots. Therefore, the proportion of the second phase affected the hardness value of the alloy. Thus, an increase in Mg content greatly improved the hardness value of the alloys.

#### 3.2.2. Tensile Properties

The effects of Mg elements on the mechanical properties of the Zn1FexMg alloys are shown in Figure 3b. Pure Zn had poor mechanical properties. Its ultimate tensile strength, yield strength, and elongation were 37 MPa, 26 MPa, and 5%, respectively. Compared with pure Zn, the UTS and yield strength (YS) of the alloys were significantly increased and there was a slight decrease in elongation. The tensile strength of the alloys increased initially and then decreased with an increase in Mg content, reaching a maximum of 157 MPa in Zn1Fe1Mg. This is because when the Mg content was ≤1 wt %, the network hard eutectic structures greatly limited the grain size of Zn crystals and they also hindered the dislocation motion of grains, thus improving the mechanical properties of the alloy. The UTS of Zn1Fe1.5Mg was less than that of Zn1Fe1Mg. The high-volume fraction brittle-coarse eutectic mixtures and massive FeZn_13_ needle-like phases greatly reduced the resistance of the alloy to crack initiation and propagation when it was stretched. However, due to the high-volume fraction hard-brittle eutectic structure phases, the elongation of all alloys was less than that of pure Zn. Casting defects also contributed to the deterioration in elongation.

**Figure 4:**
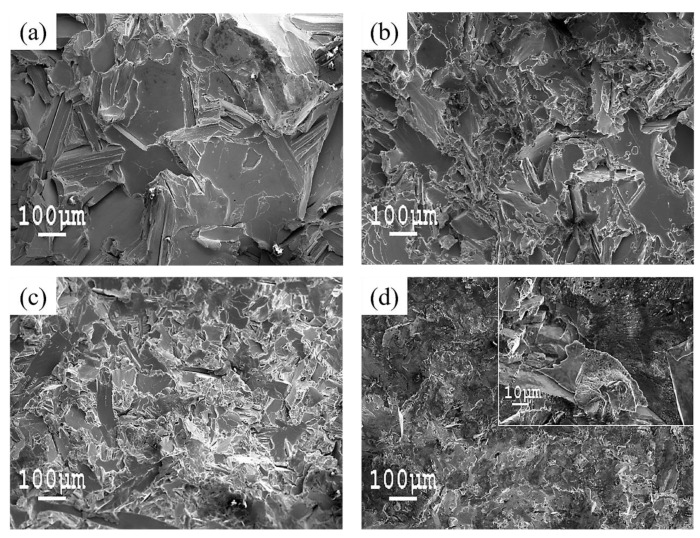
Fracture surfaces of the alloys after tensile tests: (**a**) Cast Zn, (**b**) Zn1Fe0.5Mg, (**c**) Zn1Fe1Mg, (**d**) Zn1Fe-1.5Mg.

Figure 4 illustrates fracture morphologies of the alloys after tensile testing. Figure 4a shows that pure Zn showed a brittle intercrystalline fracture without any plastic deformation. The main fracture type of the alloys shown in Figure 4b–d was transgranular cleavage fracture, which had relatively obvious river-shaped tear ridges. The tear ridges surrounded the cleavage plane and cleavage steps. There were also some intracrystalline cracks on the cleavage plane. Zn crystals were in a brittle fracture state, and the fracture was reflected as the cleavage plane. The eutectic structures surrounding the dissociation surface were in a plastic fracture state. The FeZn_13_ phase exhibited a brittle fracture, which existed as a coarse cleavage plane in the fracture morphology, and it showed a deeper contrast than the Zn dendritic grains. The area of each cleavage plane was smaller than that of pure Zn, and the fraction of the river-like pattern increased significantly, showing the characteristics of a transgranular fracture with an increase in Mg content. There was a tendency for transformation of a brittle fracture to plastic fracture with an increase in Mg content from 0.1 wt % to 1.0 wt %, and the strength and plasticity of the alloy increased accordingly. The Zn1Fe1.5Mg alloy showed completely different fracture morphology than the other alloys. The fracture occurred at the intergranular plane, and it was almost a brittle fracture. There were large amounts of eutectic particles on a flat fracture surface around the coarse Zn crystals and FeZn_13_ phases, which caused decreases in the plasticity and strength of the alloy.

Due to less solubility of Mg in Zn, which is only about 0.02–0.03 wt % [21], it is generally believed that the solid solution strengthening effect of Mg on the Zn-Mg binary alloy is less pronounced. Thus, as-cast Zn1FexMg alloys were mainly affected by grain boundary strengthening and grain refinement. The intermetallic compounds formed by the addition of Mg and Fe elements prevented slippage of the alloy crystals and controlled the dislocation, which improved the strength of the alloy; this can also explain why the plasticity of the Zn1FexMg alloy was lower than that of pure Zn. However, when the Mg content was high (Zn1Fe1.5Mg alloy), the high-volume fraction and coarse eutectic structures increased the brittleness of the alloy, which led to a decrease in the tensile strength. Zn1Fe1Mg exhibited superior mechanical properties compared to the other alloys.

### 3.3. Corrosion Properties

#### 3.3.1. Electrochemical Potentiodynamic Polarization Test

**Figure 5:**
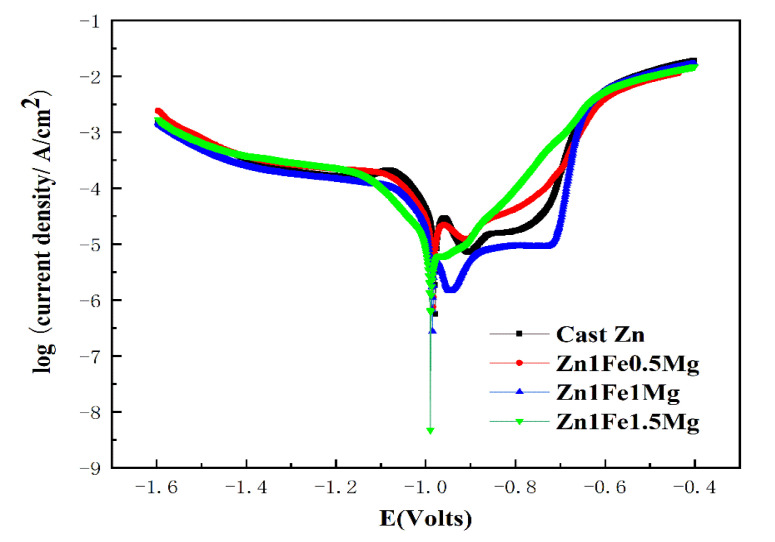
Potentiodynamic polarization analysis of tested Zn alloys in simulated body fluid (SBF) solution at ambient temperature.

The potentiodynamic curves for alloys are shown in Figure 5, and Table 3 shows the electrochemical results after potential kinetic polarization. The self-corrosion potential of the alloy in SBF at room temperature was slightly lower than that of pure Zn. Except for the Zn1Fe1.5Mg, it seems that there are two minimum current density positions in each curve. This feature represents the formation of the corrosion layer on the surface of the alloy. When the applied potential of the polarization experiment becomes higher than the self-corrosion potential of the alloy during the test, there is a potential difference between the sample and the applied potential. At this time, the sample acts as an anode and corrodes quickly, producing a dense corrosion product film layer, resulting in the phenomenon of increase in surface self-corrosion potential. Because the Zn1Fe1.5Mg alloy contains a higher content of Mg, it produces more non-compact corrosion product films containing Mg in the test, so the second minimum current density point in the corresponding curve is not obvious. This occurred because Mg_2_Zn_11_ produced by the addition of Mg caused microcell corrosion with the other phases. The electrochemical corrosion rates of alloys were significantly lower than that of pure Zn, which was different from the results of the immersion test. This may be because the alloy samples were more likely to produce insoluble oxide films than pure Zn and this led to passivation during the process of electrochemical polarization test, which caused the electrode potential to deviate from the self-corrosion potential, resulting in a decrease in the corrosion rate.

#### 3.3.2. EIS Spectra

**Figure 6:**
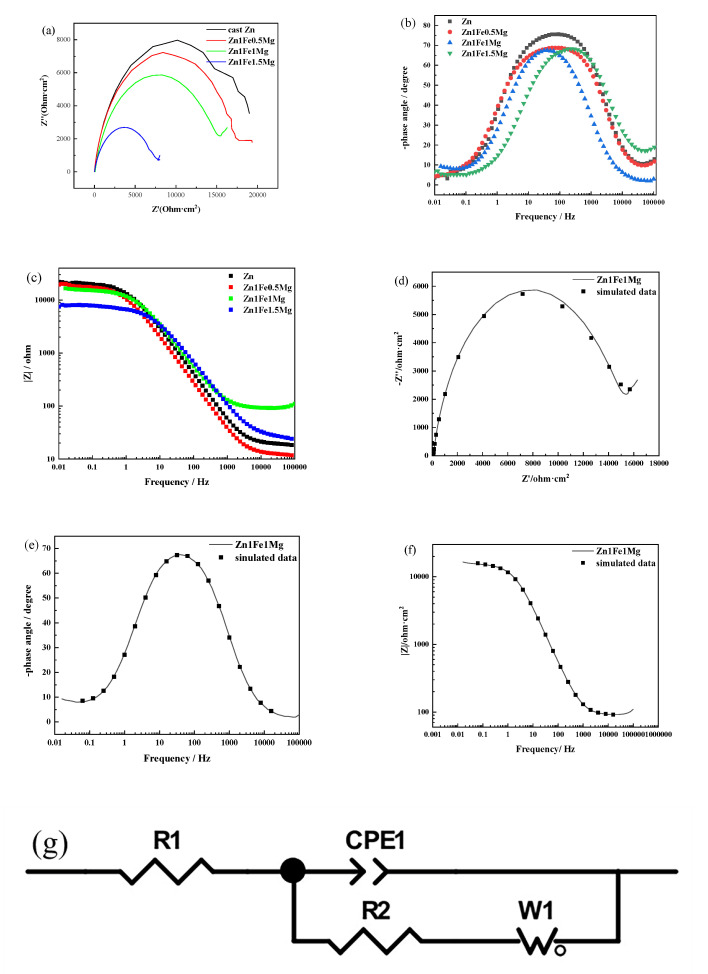
Electrochemical impedance spectroscopy (EIS) spectra of Zn-Fe-Mg in SBF solution at room temperature: (**a**) Nyquist diagram comparison and simulated results of the Zn-Fe-Mg alloy: (**b**) Bode phase angle diagram; (**c**) Bode impedance–frequency diagram; (**d**–**f**) Nyquist diagram, phase angle diagram, and impedance-frequency diagram of Zn1Fe1Mg and simulated data; (**g**) The equivalent circuit of EIS spectra for the Zn-1Fe-Mg alloy: R1 is the solution resistance; CPE1 is constant phase angle element; R2 is the charge-transfer resistance; W1 is the Warburg finite-length diffusion impedance.

The corresponding simulation curves of EIS spectra of Zn1FexMg and the fitted equivalent circuit (EC) are shown in Figure 6. The Nyquist diagrams for alloys showed the capacitive loop at all frequencies, which represented the charge transfer process and double-layer capacitance. The impedance values of Zn1FexMg were lower than those of pure Zn. With an increase in Mg content, the impedance value of the alloy decreased gradually. This indicated that the addition of Mg may cause microcell corrosion with the other phases, which reduced the corrosion resistance of the alloy. In the Bode diagram (see Figure 6b), Zn1FexMg alloys had lower peak value phase angles than pure Zn, indicating that their corrosion resistance was poor compared to that of cast Zn. Figure 6d,e shows the simulation curve of the Zn1Fe1Mg alloy. The error in fitting data was less than 5%. The EC, depicted in Figure 6g, describing a bilayer surface was used to fit the EIS spectra. Constant phase elements (CPE) were used instead of capacitors to compensate for surface inhomogeneity. This model demonstrated the general corrosion process.

#### 3.3.3. Immersion Test

**Figure 7:**
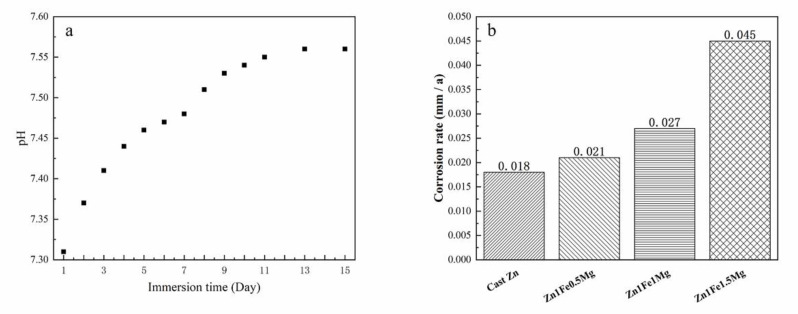
Change in the pH value of SBF solution with time after soaking the alloy (the ratio was 25 mL/1 cm^2^) (**a**) and Corrosion rates of alloys in SBF solution(**b**).

The alloy immersion experiment was performed in SBF solution to simulate corrosion of the alloy in the human body. There was an absence of gas evolution during the experiment, which is consistent with the previous research [31]. Figure 7a shows that without renewing the SBF solution, the pH value of the solution, in which the sample was soaked, gradually increased with the passage of time. This meant that corrosion of the alloy in the SBF solution caused consumption of hydrogen ions in the solution, thus making the solution alkaline. However, there is a free flow of body fluids in the human body and there is a minimal change in the pH value. Therefore, the SBF solution was frequently renewed during the immersion experiment. Figure 7b shows the average corrosion rates of alloys after 30 days of immersion in SBF solution at 37 °C. Corrosion rates of all alloys were higher than that of pure Zn, and the corrosion rate rapidly increased with an increase in Mg content. The corrosion rate of the Zn1Fe1.5Mg alloy was almost 2.5 times that of pure Zn. Rapid corrosion of alloys may have occurred due to microcell corrosion caused by the high potential difference between Mg_2_Zn_11_ and FeZn_13_. Thus, the increase in the volume fraction of the eutectic structure significantly improved the corrosion rate of the alloy.

**Figure 8:**
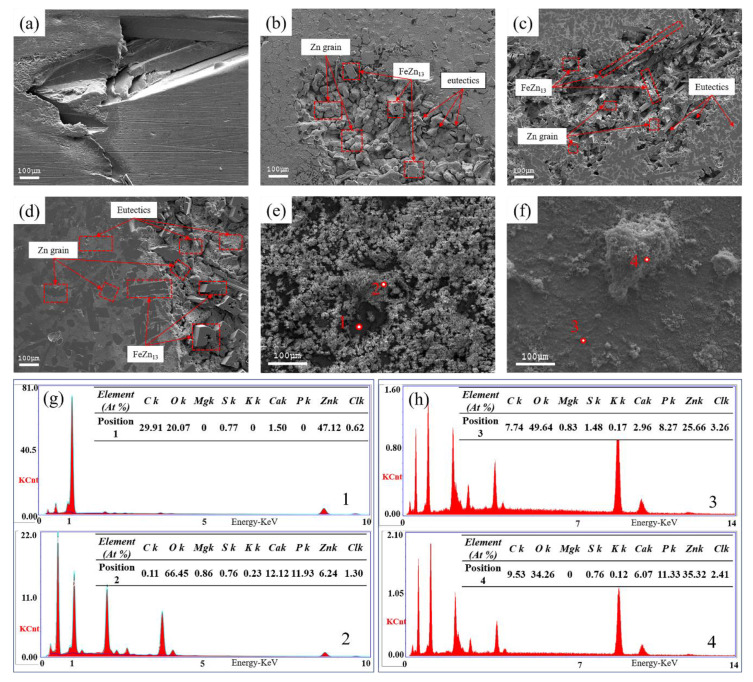
SEM micrographs of surface morphologies of (**a**) pure Zn, (**b**) Zn1Fe0.5Mg, (**c**) Zn1Fe1Mg, and (**d**) Zn1Fe1.5Mg alloys with the removal of surface corrosion products after immersion in SBF solution for 30 days; (**e**,**f**) surface morphologies of Zn1FeMg immersed in SBF for 30 days and 60 days, respectively; (**g**,**h**) EDS result corresponding to the area in (**e**,**f**), respectively.

Figure 8a–d show surface morphologies of alloys immersed in SBF solution for 30 days and then after removal of the corrosion products. It showed that the corrosion types in studied alloys were pitting corrosion and intergranular corrosion. Before the immersion test, the surfaces of tested alloys were already covered with insoluble protective films. Cl^−^ in the SBF reacted with the passivation film to disrupt the integrity of the passivation film and expose the alloy surface. The exposed surface (anode) and the passivation area (cathode) formed the active-passive corrosion cell, which caused pitting corrosion. In areas of corrosion pits, eutectic structures showed complete dissolution, causing gaps between Zn grains. Zn grains were severely corroded with obvious corrosion marks. The FeZn_13_ phase had a low corrosion degree and maintained high integrity, which prevented premature failure of the test sample. At the same time, the Mg_2_Zn_11_ phase located at the grain boundary had a lower self-corrosion potential than the other phases; therefore, the grain boundary showed high activity, which caused intergranular corrosion due to the mechanism of microcell corrosion. With an increase in Mg content, the depth of pitting pits increased and intergranular corrosion became more severe. Figure 8e,f show the surface morphology of the Zn1Fe1Mg alloy before removal of the corrosion products after SBF immersion for 30 days and 60 days. The corrosion product layer on alloys immersed for 30 days was very loose and porous, and the surface of the alloy was exposed through the micropores. Compared with the corrosion product layer on alloys immersed for 30 days, the corrosion product layer on alloys immersed for 60 days was denser, had a barely visible alloy surface, and showed growth of many white precipitates. The EDS results of corrosion products are shown in Figure 8g,h. The chemical elements of corrosion products were mainly Zn, Ca, P, O, C, Cl, S, and a small amount of Mg and K. The results of EDS combined with the Pourbaix diagrams of the Zn-x-H2O (x = p, s, c, cl, etc) system [11] indicated that the corrosion products comprised zinc phosphates, zinc carbonates, zinc hydroxide, and calcium phosphate.

#### 3.3.4. Corrosion Model of Zn1FexMg

**Figure 9:**
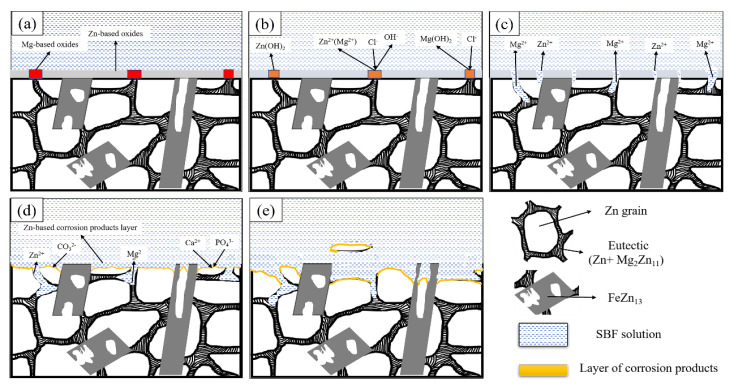
Schematic diagram of in vitro biocorrosion of the Zn1FexMg alloy in simulated body fluids: (**a**–**e**) are the sequence from the beginning of corrosion to the detachment of a small part of the Zn Matrix.

The effect of Mg elements on the corrosion mechanism of the alloy can be explained by the interaction between the eutectic structure, FeZn_13_ phase, and the Zn crystals. Based on the above results and related literature, an in vitro corrosion model of the Zn1MgxFe alloy-liquid interface was proposed, as shown in Figure 9. The corrosion behavior of the alloy in SBF solution can be inferred as follows:

(1) The alloy sample had oxide film protective layers before immersion, which mainly included ZnO, MgO, and a small amount of iron oxide (see Figure 9a). MgO film layers were loose and porous. Thus, electrolytes in SBF solution were exposed to the surface of the studied alloys through micropores in the MgO film layers, causing pitting corrosion of the alloy surface. The Mg_2_Zn_11_ had a lower self-corrosion potential than the other structures. Thus, grain boundaries could be corroded preferentially, and an increase in Mg element aggravated this phenomenon. Dissolution of the eutectic structure allowed the fresh SBF solution to make contact with the Zn grains, causing corrosion of the matrix (see Figure 9b). Corrosion of the zinc matrix was accompanied by reduction in the zinc solution in the anode and oxygen in the cathode, and the reactions were as follows:
  anodic reaction:
    $$Zn\left(Mg\right)\to Zn^{2+}+2e$$
  cathodic reaction:
    $$2H_{2}O+O_{2}+4e\to 4OH^{-}$$
  overall reaction:
    $$2Zn\left(Mg\right)+2H_{2}O+O_{2}\to 4Zn\left(Mg\right){\left(OH\right)}_{2}$$
(2) The eutectic structures were corroded very quickly, which made deposition and concentration of Mg(OH)_2_ on the alloy surface difficult. Thus, Mg(OH)2 could not provide an effective barrier to further corrosion protection. The zinc hydroxide film covered the surface of the alloy, which could effectively prevent the dissolution of Zn. The results of EDS (see Figure 8g,h) showed that almost all corrosion products were enriched in Cl element. However, Cl^−^ in the SBF solution disrupted the balance between the formation and dissolution of zinc hydroxide and reacted with it to form chlorohydroxy compounds. Since the self-corrosion potential of the FeZn_13_ phase was higher than that of the other phases, the areas adjacent to the FeZn_13_ phase corroded faster than those at other positions due to high potential difference (see Figure 9c). The reaction for formation of chlorohydroxy compounds was as follows:
  $$6Zn{\left(OH\right)}_{2}+ZnCl_{2}↔7ZnCl_{2}{\left(OH\right)}_{12}\left(s\right)$$
(3) As the corrosion process continued, corrosion areas gradually extended from the position of the eutectic structure to the Zn matrix. Deep corrosion pits were formed (see Figure 9d). The EDS result shows a high content of Zn, C, O element and a few Ca in position 1 (see Figure 8e), indicating the formation of zinc carbonate, which can effectively improve the corrosion resistance of Zn by promoting the formation of zinc hydroxide [12]:
  $$5Zn^{2+}+2HCO_{3}^{-}+6H_{2}O\to Zn_{5}{\left(OH\right)}_{6}{\left(CO_{3}\right)}_{2}\left(s\right)+8H^{+}$$
  $$2H^{+}+2HCO_{3}^{-}+H_{2}O+5ZnO\left(s\right)\to Zn_{5}{\left(OH\right)}_{6}{\left(CO_{3}\right)}_{2}\left(s\right)$$
(4) With continuous horizontal and vertical expansion of the pitting corrosion area, the corrosion product layer became denser. The presence of carbonate in corrosion products promoted the deposition of phosphate. The discovery of Ca, Zn and P, O elements in the EDS results proved the existence of phosphate. The presence of zinc phosphate could promote the proliferation of bone cells. According to the following reaction, solid surface films could be formed in an aqueous solution containing phosphate, which could significantly inhibit the dissolution of Zn [31]:
  $$6Ca^{2+}\left(Zn^{2+}\right)+2HPO_{4}^{2-}+2H_{2}PO_{4}^{-}+6OH^{-}\to 2{\left(Ca,Zn\right)}_{3}{\left(PO_{4}\right)}_{2}+6H_{2}O$$
(5) As the alloy continued to corrode, the corrosion products gradually increased. Even small parts of Zn grains peeled off due to complete dissolution of the eutectic structures around the Zn grains. Relatively complete FeZn_13_ phases were present and they still provided support (see Figure 9e).

The corrosion rates of alloys could simply indicate the amount of Zn element released, which helps to infer the potential toxicity of the implant in the human body from a biocompatibility viewpoint. We assume that a HA5.0 bone screw was made using Zn1FexMg with a working part size of φ5 × 50 mm. The corrosion rate of the screw in a neutral environment was about 0.021–0.045 mm/a, as indicated in Figure 7b. Thus, the average daily Zn absorption from this screw can be calculated to be about 0.66–1.4 mg/d, which was far below the toxicity limit for Zn (100–150 mg/d).

## 4. Conclusions

Zn-1Fe-xMg alloys comprised primary dendritic Zn grains, FeZn_13_ prismatic structures, and the eutectics of Zn + Mg_2_Zn_11_. With an increase in Mg content, the fraction of eutectic structures increased gradually; this made the Zn crystals more uniform and finer, but it also caused slight coarsening of FeZn_13_. Hardness, yield strength, and ultimate tensile strength of the alloys increased significantly with an increase in Mg content. The corrosion behavior evaluation by the potentiodynamic polarization test, EIS impedance test, and immersion test showed that the increase in Mg content exacerbated microcell corrosion between different phases, which reduced the impedance and self-corrosion potential of the alloys, thus improving the corrosion rate of alloys in SBF solution. Zn1Fe1Mg possessed superior comprehensive mechanical properties (its hardness, tensile strength, yield strength, and elongation values were 105 HB, 157 MPa, 146 MPa, and 2.3%, respectively) and had an appropriate corrosion rate (0.027 mm/a), thus revealing that it is a preferred candidate for biodegradable implant material.

## Figures and Tables

**Table 1:** The actual components of alloys measured by Inductively Coupled Plasma Optical Emission Spectrometry (ICP-OES).

Number	Symbol	Nominal Composition (wt %)	Measured Composition (wt %)				
			Zn	Fe	Mg	Cu	Sn
#1	Zn	Zn	99.99	<0.001	<0.001	<0.002	<0.001
#2	Zn1Fe0.5Mg	Zn-1wt %Fe-0.5wt %Mg	98.43	0.93	0.6	<0.002	<0.001
#3	Zn1Fe1Mg	Zn-1wt %Fe-1wt %Mg	97.81	0.9	1.25	<0.002	<0.001
#4	Zn1Fe1.5Mg	Zn-1wt %Fe-1.5wt %Mg	97.12	0.98	1.85	<0.002	<0.001

**Table 2:** Grain sizes of alloys and FeZn13 phase size of alloy samples.

Samples	Pure Zn	Zn1Fe0.5Mg	Zn1Fe1Mg	Zn1Fe1.5Mg
Size of Zn grain	312.54 μm	38.80 μm	22.08 μm	38.04 μm
Section size of FeZn_13_		36.13 μm	38.10 μm	62.65 μm

**Table 3:** Fitted results of polarization curves in Figure 5: Ecorr is Corrosion Potential; icorr is corrosion current density; C.R is the corrosion rate.

Alloys	E_corr_(V vs. SCE)	i_corr_(mA/cm^2^)
Cast Zn	−0.97	5.44 × 10^−5^
Zn 1Fe0.5Mg	−0.98	2.44 × 10^−5^
Zn1Fe1Mg	−0.98	4.18 × 10^−5^
Zn1Fe1.5Mg	−0.99	1.54 × 10^−5^
